# Genome Characterisation of Invasive *Haemophilus influenzae* in Pregnancy: The Noticeable Placental Tissue Tropism Is Distributed across the Species Rather Than Linked with Capsulation or Particular Clones

**DOI:** 10.3390/pathogens12111345

**Published:** 2023-11-13

**Authors:** Niels Nørskov-Lauritsen, Rajesh Mohey, Dennis S. Hansen, Liv Duus, Mohammad R. Khalil, Stella J. Wilfred, Stine Y. Nielsen

**Affiliations:** 1 Department of Clinical Microbiology, Odense University Hospital, DK-5000 Odense, Denmark; niels.norskov-lauritsen@rsyd.dk; 2 Department of Clinical Microbiology, Aarhus University Hospital, DK-8200 Aarhus, Denmark; livmardu@rm.dk; 3 Department of Medicine, Region Hospital Viborg, DK-8800 Viborg, Denmark; r.mohey@rm.dk (R.M.);; 4 Department of Clinical Microbiology, Copenhagen University Hospital, DK-2730 Herlev, Denmark; dennis.schroeder.hansen.01@regionh.dk; 5 Department of Obstetrics and Gynecology, Lillebælt Hospital, DK-6000 Kolding, Denmark; mohammad.khalil@rsyd.dk; 6 Department of Clinical Microbiology, Lillebælt Hospital, DK-7100 Vejle, Denmark; 7 Department of Biomedicine, Aarhus University, DK-8000 Aarhus, Denmark

**Keywords:** *Haemophilus influenzae*, whole-genome sequencing, pregnancy, infection, abortion, neonatal, bacteraemia

## Abstract

Pregnancy is associated with a 5–26 times increased risk of invasive *Haemophilus influenzae* infection and subsequent adverse pregnancy outcomes. Incidence rate and outcome are published in some regions, but the characterisation of bacterial isolates is limited. We performed comparative genomic analyses of isolates from 12 pregnancy-associated cases, cultured from maternal bacteraemia in pregnancy (nine), postpartum bacteraemia (one), neonatal bacteraemia (one), and placental tissue (one). In two bacteraemia cases, identical isolates were also cultured from cervical swabs. Eight cases occurred early in pregnancy (gestational week 7–26), and seven of them resulted in miscarriage or neonatal death. All bacterial genomes were devoid of capsule loci, and they were evenly distributed in the major phylogenetic group I of the species. The conspicuous tropism of *H. influenzae* for pregnancy and placental tissue is associated with the species rather than specific clonal subtypes.

## 1. Introduction

Childhood vaccination against *Haemophilus influenzae* serotype B all but eradicated meningitis caused by the bacterium, and focus shifted to less severe but far more prevalent infections caused by unencapsulated variants, such as acute otitis media and exacerbation in chronic obstructive pulmonary disease [1,2,3]. Nevertheless, invasive *Haemophilus influenzae* remains an important cause of morbidity and mortality in young children, older adults, and groups with certain dispositions. Overall incidence rates in Active Bacterial Core (ABC) surveillance catchment areas of the US in 2009–2015 were 1.7 per 100,000 person years, but 8.5 for infants < 1 year and 6.3 for people ≥ 65 years [4].

Only recently has pregnancy been recognised as a specific disposition for infection with *H. influenzae*. European surveillance data on invasive *H. influenzae* in 1996–2006 disclosed that the incidence of unencapsulated strains had surpassed that of *H. influenzae* serotype B and was associated with higher case fatalities [2]. An interesting observation was that invasive *H. influenzae* disease was more common in males than in females overall, but the opposite was true for those aged 18–44 years. The observation was extended to Iceland, where pregnancy was associated with a markedly increased susceptibility to invasive *Haemophilus* infections (relative risk ratio of 26) compared with non-pregnant women [5]. Public health data from England and Wales in 2009–2012 also linked female vulnerability to pregnancy, which was associated with a 17-fold increase in incidence compared to non-pregnant women [6]; of 47 infections in the first 24 weeks of pregnancy, 44 were associated with foetal loss.

From the US ABC catchment areas 2008–2019, the risk of invasive *H. influenzae* infection was six times higher in pregnancy and postpartum compared to non-pregnant women. In contrast to non-pregnant women of childbearing age, pregnant and postpartum women were younger and healthier; all had bacteraemia, and none died. There were 59 pregnant and 46 postpartum cases, and the outcome of 52 pregnancies was spontaneous abortion or stillbirth [7].

*H. influenzae* diseases associated with pregnancy encompass maternal bacteraemia in pregnancy and postpartum, chorioamnionitis, and neonatal infection. A distinct epidemiology was reported from New Zealand, consisting of 52 maternal and/or neonatal invasive *H. influenzae* infections during 2008–2018 [8]. While infant bacteraemia was detected in 15 of 16 neonatal infections, bacteraemia was only documented in 13 of 38 maternal infections, where the predominant culture-positive specimen was placental tissue or products of conception. *H. influenzae* was isolated from amniotic fluid specimens of 8 of 110 consecutive women with preterm premature rupture of membranes in Chile in 1992–1998 [9].

Neonatal invasive *H. influenzae* infections are almost exclusively bacteraemia cases with isolation of the pathogen within 24 h of birth. Maternal bacteraemia is not the principal, documented source of neonatal infection, as it is only observed in 6–15% of cases [7,8,10].

Thus, the epidemiology and outcome of invasive *H. influenzae* infection in pregnancy are described, but the characterisation of the aetiologic agent beyond capsulation status is scarce. Ten neonatal strains from Italy in 2009–2015 belonged to discrete multilocus sequence types (STs) via PCR and Sanger sequencing, indicating a high degree of genetic diversity [11]. From the recent US ABC data, isolates from all five mother-infant pairs were identical via whole genome sequence but belonged to five separate STs [7].

The aim of this study was to characterize the genome of invasive isolates of *H. influenzae* in order to disclose specific clonal subtypes or clusters associated with pregnancy.

## 2. Materials and Methods

### 2.1. Cases

A noticeable case (#6) sparked clinical concern, and five other contemporaneous cases were rapidly identified. *H. influenzae* isolates associated with pregnancy in 2018–2021 were collected from four Danish departments of Clinical Microbiology. Previously collected isolates from one of the participating departments were also included. Permission to extract information from patient records was obtained. Clinical information on admission and the outcome of pregnancy is presented in Table 1. All women presented with abdominal pain, fever, and elevated markers of infection. *H. influenzae* was isolated from blood cultures. For case #4, *H. influenzae* was also cultured from a vaginal swab.

Case #1 developed clinical signs of infection in late pregnancy, was admitted in gestational week 40, and delivered a healthy infant three days later.

For the five other cases, the outcome was a missed abortion or miscarriage. The time from sampling of blood culture to missed abortion or miscarriage was between one and three days.

Case #2 presented with fever and abdominal pain in gestational week 8; due to missed abortion, suspected bleeding and sepsis, an evacuation followed by laparoscopy was performed.

For Cases #3, #4, and #5, the foetus was alive when the women presented with abdominal pain at gestational weeks 15, 16, and 7, respectively, but all three women miscarried one day later.

Case #6 carried a live foetus upon admission at gestational week 6 but developed sepsis, and the evacuation of a missed abortion was performed.

To expand our investigation of invasive *H. influenzae* in pregnancy, isolates from six additional cases were included in the study. Case #7 is recent, while isolates from cases #8–12 were stored in one of the participating microbiology departments during 2010–2014. Permissions do not cover these cases, and patient records were not consulted. Rudimentary clinical information (included in Table 1) is reconstructed from descriptions accompanying samples and from stored laboratory messages conveyed to requisitioning physicians. Among the additional six cases, there were three healthy infants, one neonatal death, and one stillbirth. In one case, the outcome of the pregnancy was unknown (case #11).

For Case #7, bacteraemia and vaginal discharge occurred five days after the delivery of a healthy infant at term. *H. influenzae* was cultured from blood and a vaginal swab.

For Case #8 there was bacteraemia of a premature infant (gestational week 26), blood sampled on the day of birth, and neonatal death from sepsis within 48 hours.

For Case #9, there was bacteraemia of a woman with chorioamnionitis at gestational week 25. Positive blood cultures were sampled the day before and on the day of the caesarean section. Infant was healthy at delivery and without signs of septicaemia for two weeks.

For Case #10 there was bacteraemia of a pregnant woman, with stillbirth one week after preterm, premature rupture of membranes at gestational week 24.

For Case #11 there was bacteraemia of a pregnant woman at an unknown gestational week, and hospitalisation was needed due to symptoms of endometritis. The patient was discharged prior to dispatchment of the final laboratory report.

For Case #12, a culture (swab) was obtained from foul-smelling placenta after caesarean section at an unknown gestational week. Infant was healthy, and the mother had a prompt recovery after antimicrobial treatment.

Thus, the combined case series comprises 9 cases of bacteraemia during pregnancy (including two with chorioamnionitis verified via vaginal culture of the pathogen), one postpartum bacteraemia, one neonatal bacteraemia, and one invasive *H. influenzae* infection documented via culture from a foul-smelling placenta. The outcome was miscarriage in six cases (gestational age between 6 and 24 weeks) and one stillbirth (gestational week 24). One premature infant (gestational week 26) died from septicaemia two days after birth. There were at least four healthy infants, and all women recovered. All cases received relevant antimicrobial treatment.

### 2.2. Isolates, Whole Genome Sequencing and Bioinformatics Analysis

Isolates were identified to species level with matrix-assisted laser desorption/ionisation time-of-flight (MALDI-TOF), Bruker, Bremen, Germany, and stored at −80 °C at the participating departments. For the present study, isolates were thawed and propagated on chocolate agar plates. The extraction of DNA, library preparation, Illumina sequencing, and assembly of contigs of isolates from cases #1 to #7 were performed as previously described [12]. Isolates from cases #8 to #12 were sequenced with long-range technology. Libraries were prepared using Rapid Barcoding Kit 96 (SQK-RBK110.96), loaded onto a MinIONFlow cell (R9.4.1) and sequenced on a MinION instrument using MinKNOW 23.04.5 (all from Oxford Nanopore Technologies plc, Oxford Science Park, United Kingdom. Basecalling was performed with Guppy v. 6.5.7 using the high-accuracy model. Basecalled reads were demultiplexed and adaptors trimmed with porechop v. 0.2.4. The trimmed reads were assembled into contigs with Flye v. 2.9 and polished with medaka v. 1.5.0.

Prokka-annotation and identification of core genes with Roary, calling of SNPs, and analysis of evolutionary distances with MEGA X were performed as previously described [12]. Genomes were classified at the Genome Taxonomy Database using GTDB-tk [13,14]. *H. influenzae* STs were called by submission of genomes to PubMLST [15], and virulence genes were searched in the Virulence finder database [16].

## 3. Results

### Genomic Characterisation of Causative Agents

Fourteen *H. influenzae* isolates from 12 cases were genome sequenced and compared with reference strains. Study strains were bacteraemia isolates, except case #12, which was cultured from a foul-smelling placenta; in cases #4 and #7, *H. influenzae* was also cultured from cervical swabs. All isolates were categorised as non-typeable *Haemophilus influenzae* (NTHi), as revealed via standard slide agglutination serotyping.

**Figure 1:**
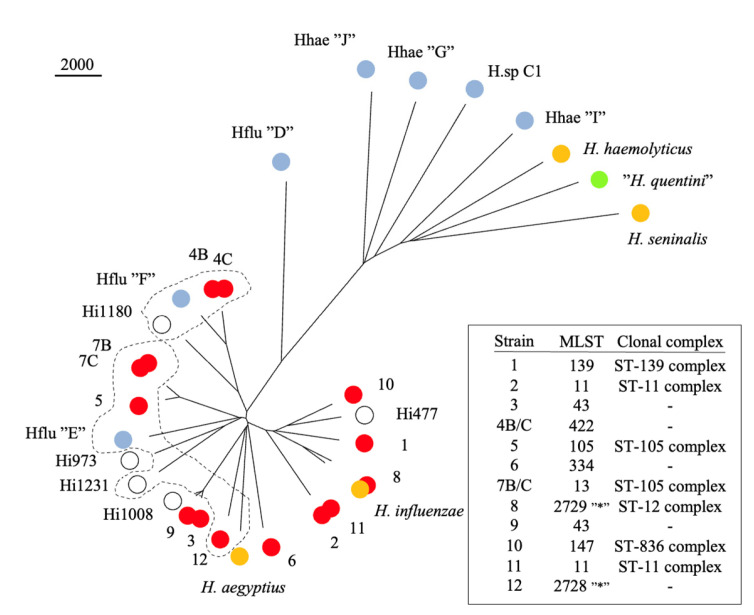
Neighbour-joining dendrogram of 425 *Haemophilus* core genes depicting the relationship the study strains and the taxonomy of closely related species within the genus. Red circles, 14 invasive *H. influenzae* strains from 12 pregnancies (B and C designate blood and cervix from the same case); orange circles, type strains of validly published species names; open circles, reference strains of *H. influenzae* phylogenetic group I (see text for details); light blue circles, fastANI reference sequences from GTDB representing putative novel species; green circle, reference strain of “*Haemophilus quentini*” (not validly published name). Freeform shapes encircle putative species *Haemophilus influenzae* E and *Haemophilus influenzae* F. Alignment is based on 351,171 nt; bar represents 2,000 residue substitutions. The asterisks denote new MLST sequence types described in this study.

According to EUCAST phenotypic criteria, all study strains were susceptible to ß-lactam antimicrobials, with the exception of the two isolates from case #4 that were resistant to ampicillin, susceptible to cephalosporins, and produced ß-lactamase, as revealed with a nitrocefin test. Genome sequence analysis revealed the presence of *bla*_TEM-1_ in these two isolates; TEM-1 is a narrow-spectrum ß-lactamase that hydrolyzes the penicillin class of ß-lactams [17]. None of the study strains encoded capsulation genes (*bex*A–D) or a functional CRISPR-cas system. The study strain sequences were compared with type strains of *H. influenzae, Haemophilus aegyptius*, *Haemophilus haemolyticus,* and *Haemophilus seminalis*, with five reference unencapsulated isolates [18], and with selected fastANI reference sequences of putative new species from the Genome Taxonomy Database (GDTB) [13,14] (Figure 1).

All study isolates belonged to phylogenetic group I, as defined by Meats and co-workers in the original description of the multilocus sequence typing (MLST) scheme of *H. influenzae* [19]. Paired isolates (blood and cervix) from cases #4 and #7 were identical (zero single nucleotide polymorphism (SNP) in 425 core genes); otherwise, the isolates were broadly distributed in phylogenetic group I. The closely related isolates #2 and #11 (both isolates of ST 11) were separated using 24 SNPs; however, they were identified in different maternity wards five years apart. Isolate #9 differed from the international reference isolate Hi1008 using 584 SNPs (both isolates of ST 43).

The predominance of phylogenetic group I and the uniform distribution of isolates within the group mimic a recent, nation-wide characterisation of *H. influenzae* cultured from various specimens on a single day in Denmark, where all 62 isolates belonged to phylogenetic group I [20]. Freeform shapes in Figure 1 depict the borders of the putative species “*Haemophilus influenzae* E” and “*Haemophilus influenzae* F”, as defined by pairwise Average Nucleotide Identity (ANI). The conspicuous delineation of “*Haemophilus influenzae* E” underscores the discrepant results obtained via neighbour-joining alignment of shared genes vs. pairwise ANI. If the comparison is restricted to sequences from phylogenetic group I, 773 core genes spanning 657,792 nt can be identified, but reference strain Hi973 (not part of “*Haemophilus influenzae* E”) is still positioned on a common branch with strain Hi1231 (included in “*Haemophilus influenzae* E” by ANI).

In addition to *H. influenzae* phylogenetic group I, Figure 1 encompasses “*Haemophilus influenzae* D” as a representative of phylogenetic group II (comprising rarely detected lineages including capsulated serotypes e and f [19]), plus reference sequences of *Haemophilus haemolyticus*, *Haemophilus seminalis*, “*Haemophilus quentini*”, and related genomospecies defined by ANI. Nucleotide accession numbers of the study and reference strains are given in the Data Availability Statement below.

## 4. Discussion

Almost 100 years have passed since Margaret Pittman described the type specificity of *H. influenzae* [21]. The presence of polysaccharide capsular antigen provides the basis for serotype designations, and although most strains are unencapsulated, the dreadful association of serotype B with childhood meningitis motivated a biased focus on capsulate strains (serotypes a–f). Thus, the majority of strains detected in the clinical microbiology laboratory was designated as non-typeable due to the lack of a capsule. In 2003, MLST of *H. influenzae* [19] revealed a division into two phylogenetic groups, where group II encompassed serotypes e and f, as well as other rarely encountered lineages (represented by ”Hflu D” in Figure 1). Only strains of phylogenetic group I were detected in a recent nationwide characterisation of *H. influenzae* [20], and this core of the species has not been validly separated into lineages. By genome sequencing, De Chiari and co-workers identified a discrete population structure and separated phylogenetic group I into five clades [18], whereas GTDB has suggested three new species (*Haemophilus influenzae* D–F) [13,14].

Forty years ago, variant strains of *H. influenzae* isolated from the genitourinary tract were reported from Canada [22]. Subsequently, Quentin and co-workers typed urogenital, maternal, and neonatal isolates of *H. influenzae* and described the genital specificity of *H. influenzae* biotype IV (negative for tryptophanase and positive for urease and ornithine decarboxylase) [23]. Later, characterisations have placed the cryptic genospecies (“*Haemophilus quentini*”) close to *H. haemolyticus* [24,25], which is corroborated via whole genome sequencing (Figure 1).

Although 1 of 10 neonatal invasive *H. influenzae* isolates from Italy in 2009–2015 and 1 of 8 amniotic fluid isolates from Chile in 1992–1998 belonged to “*Haemophilus quentini*” via 16S rRNA gene sequencing [9,11], it is *H. influenzae* in the taxonomically narrow sense that is associated with invasive infections in pregnancy. All 12 cases belonged to phylogenetic group I of *H. influenzae*. The bacterial species concept has been revised and challenged in the genomic era [26,27]. An ANI of approximately 95% is widely used for delineation of genomospecies, and by using this breakpoint, *H. influenzae* may be divided into five genomospecies (Figure 1). However, in the context of pregnancy-associated infection, the distinction between genomospecies has little clinical significance. Although most of the isolates from our cases were related to the type of strain of the species, some of the isolates clustered with the putative species “*Haemophilus influenzae* E” and “*Haemophilus influenzae* F”. The wide distribution of isolates within phylogenetic group I testifies to the general pathogenic potential of *H. influenzae*. Isolates of phylogenetic group II (or “*Haemophilus influenzae* D”) were not detected in the current study.

The spectrum of pregnancy-associated infections includes maternal bacteraemia, chorioamnionitis, postpartum infections, and neonatal infections. Maternal and neonatal septicaemia can be accurately extracted from public health data, but the underlying cause of miscarriage or preterm delivery can go unnoticed, as exemplified by our case #12. The diagnosis relies on the microbiological investigation of placental and foetal samples, and a significant proportion of *Haemophilus*-related preterm delivery or pregnancy loss may go unrecognised. A study from Scotland in 2017–2018 reviewed all post-mortems at <24 weeks gestation with histologically proven acute chorioamnionitis on placental histology; *H. influenzae* accounted for 20% of infections associated with early pregnancy loss prior to week 24 [28]. The inconsistent definition, or reporting, of invasive *H. influenza* infections in pregnancy is apparent from the contrasting numbers of bacteraemia, from 100% in the US ABC catchment areas 2008–2019 [7] to 34% in the New Zealand study [8].

The present case series springs from clinical concerns, but certain epidemiological trends are recognised. Maternal invasive *H. influenzae* infection during the first 24 weeks of pregnancy is associated with a high rate of foetal loss, while stillbirth is rare in the second half of pregnancy. Only a single neonatal bacteraemia case is included, but our case #8 supports the recognition of early-onset septicaemia with a substantially increased risk among preterm neonates, where maternal bacteraemia is rarely documented, and with a grave outcome [7,8,10].

An essential clinical question relates to the port of entry of the pathogen. One possible hypothesis is that sexually acquired vagino-cervical *H. influenzae* infection is the proximate cause of invasive infections in pregnant women [8]. There are some uncertainties about this hypothesis. Detection of *Haemophilus* spp. is common in male urethral infections [29,30,31,32], but *H. influenzae* is rare, the predominant species by culture being *Haemophilus parainfluenzae*. *H. seminalis* was recently named pertaining to the isolation of two strains from human semen. The species is related to *H. haemolyticus* (Figure 1). Comparative genomic analyses have recently identified *H. seminalis* cultured from other locations, including urine and cerebrospinal fluid [33]. However, it is unlikely that strains of *H. seminalis* were previously misidentified as *H. influenzae*, as the functional heme biosynthesis pathway present in *H. seminalis* provides independence of hemin (X-factor), which is a classical phenotypic trait of *H. parainfluenzae*.

A study dating back to the 1990s reported a very low genital carrier rate of *H. influenzae* among pregnant women (0.18%) [34], and the low rate was recently corroborated by the absence of genital carriage of *H. influenzae* among 510 pregnant women in Italy [35]. These studies are linked to the culture of a fastidious organism, which may be overgrown by other species of the vaginal microbiota. A recent investigation assessed the *H. influenzae* vaginal carrier rate in non-pregnant reproductive-age women via quantitative PCR of *hpd* and disclosed a carrier rate of 4.4% [36]. The study used a primer/probe set validated by the Centre for Disease Control and Prevention, Atlanta, US, for the diagnosis of meningitis and confirmed positive cases via Sanger sequencing of amplification products. It is questionable whether this assay can discriminate between *H. influenzae* and closely related, naturally competent species in a complex microbiota and to what extent the DNA traces represent viable bacteria, but the data suggest a possible presence of *H. influenzae* in the vaginal microbiota at a low rate. If a mixed microbiota on rare occasions can pass the firm and closed cervix of pregnancy, the subsequent development of a monospecies infection does indicate that *H. influenzae* possesses a specific tropism for placental tissue.

Another possible hypothesis of port of entry is that short and asymptomatic *H. influenzae* bacteraemia following minor bruises from, e.g., toothbrushing precedes placental infection. This would mimic the experience of infective endocarditis, where certain oral *Streptococcus* species cause the malady due to a specific tropism for dilapidated heart valves, sometimes without any signs of oral or dental abscess. However, *H. Influenzae* resides in the nasopharynx, not in the oral cavity [25]. Case #10 is an example of maternal bacteraemia concomitant with stillbirth one week after premature rupture of membranes, but the cause of rupture, time of infection, and port of entry are unknown. Irrespective of the port of entry, the tropism for placental tissue is an integral part of the disease, as shown by the gloomy outcome for the foetus if maternal bacteraemia occurs during the first 24 weeks of pregnancy (Table 1).

In the post-*H. influenzae* type b (Hib) vaccine era, focus has shifted to infections with unencapsulated *H. influenzae*, often designated non-typable *H. influenzae* or NTHi [1]. A mean annual notification rate of invasive *H. influenzae* disease of 0.6 cases/100,000 population in Europe 2007–2014 is not distressing, but a rate of 23.6/100,000 among patients < 1 month of age is worrisome [37]. Invasive *H. influenzae* infection during pregnancy is an under-recognised syndrome in light of its frequency and severity. Experimental studies are required to elucidate the specific interaction between *H. influenzae* and the products of conception.

## Figures and Tables

**Table 1:** Characteristics, clinical findings, and pregnancy outcomes among 12 cases infected with *H. influenzae*.

	Age	Symptoms, Paraclinical Findings, and Gestational Age upon Admission	Outcome	Time from Admission (Blood Culture Taken) to Outcome or Delivery Days (d)
**Patient #1**	18	Abdominal pain, decreased foetal movement, increased amounts of discoloured vaginal discharge WBC: 12.3; CRP: 13 US: IUGR GA: 40 w + 2 d	Healthy newborn	3 d
**Patient #2**	37	Abdominal pain, fever WBC: 8.9; CRP: 13 US: missed abortion GA: 8 w + 4 d	Missed abortion Evacuation Laparoscopy	3 d
**Patient #3**	23	Abdominal pain, fever WBC: 15.4; CRP: 58 US: normal fetus, alive GA: 15 w + 1 d	Miscarriage Pathology report: placenta with chorioamnionitis and inflammatory response	1 d
**Patient #4**	37	Abdominal pain, fever WBC: 13.5; CRP: 108 US: normal fetus, alive GA: 16 w + 5 d	Manual removal of placenta	1 d
**Patient #5**	31	Abdominal pain, fever WBC: 9; CRP: 9.5 US: normal fetus, alive GA: 7 w	Miscarriage Evacuation	2 d
**Patient #6**	32	Abdominal pain, fever, vaginal bleeding WBC: NA; CRP: NA US: normal fetus, alive GA: 6 w + 5 d	Missed abortion Evacuation	1 d
**Patient #7**	NA	Abdominal pain, fever, vaginal bleeding WBC: 7.8; CRP: 102 US: endometritis GA: NA	Healthy newborn Fever and vaginal discharge 5 days postpartum	Postpartum
**Patient #8**	NA	GA: 26 w	Neonatal death from sepsis 48 h postpartum	NA
**Patient #9**	NA	Fever GA: 25 w	Healthy newborn Chorioamnionitis	1 d
**Patient #10**	NA	Fever GA: 24 w	Stillbirth one week after PPROM. Adherence of placental tissue	NA
**Patient #11**	NA	NA	Unknown Endometritis	NA
**Patient #12**	NA	NA	Healthy newborn Foul-smelling placenta	0 d

WBC: White Blood Cell count (10^9^/L); CRP: C-reactive protein (mg/L); US: ultrasound; IUGR: Intrauterine growth retardation; GA: gestational age (weeks + days); NA: not available; and PPROM: preterm, premature rupture of membranes.

## Data Availability

Whole Genome Sequences from project PRJNA997135 are deposited at DDBJ/ENA/GenBank under accession numbers JAUPHH000000000-JAUPHP000000000. Genomes with previously unknown ST are also deposited in PubMLST.org (case #8 and #12 as well as Id 25072 and 25073, respectively). Accession numbers of reference type strains are as follows: *Haemophilus influenzae* strain NCTC 8143^T^, NZ_LN831035.1; *Haemophilus aegyptius* strain NCTC 8502^T^, LS483429.1; *Haemophilus haemolyticus* strain CCUG 12834^T^, NZ_LYCK01000001.1; and *Haemophilus seminalis* strain SZY H1^T^, NZ_VCED01000001.1. Reference sequence of “*Haemophilus quentini*” strain K068 (not validly published), MDJB01000001.1. GTDB putative species types “*Haemophilus influenzae* D”¸ NZ_LS483411.1; “*Haemophilus influenzae* E”, NZ_QWMH01000001.1; “*Haemophilus influenzae* F”, NZ_MZHA01000001.1; “*Haemophilus haemolyticus* G”, NZ_AFQO01000022.1; “*Haemophilus haemolyticus* I”, NZ_CP031243.1; “*Haemophilus haemolyticus* J”, NZ_QQKA01000001.1; and “*Haemophilus* species C1”, NZ_LDVZ01000082.1. Representative strains of the five *H. influenzae* phylogenetic group I clades of De Chiara and co-workers are extracted from [18] as previously described [20].
